# Supplementary material for: Prevalence of chronic pulmonary aspergillosis along the continuum of pulmonary tuberculosis care: A protocol for a living systematic review and meta-analysis
Source: PLoS One. 2023 Dec 15;18(12):e0294634. doi: 10.1371/journal.pone.0294634 (PMC10723662; doi:10.1371/journal.pone.0294634)
Supplement: Attachment — Submitted filename: Response to Reviewers PLoS ONE.docx [file pone.0294634.s002.docx]

**Response to Reviewers**

PONE-D-23-30285

Prevalence of chronic pulmonary aspergillosis along the continuum of pulmonary tuberculosis care: a protocol for a living systematic review and meta-analysis

PLOS ONE

Dear Dr Aleksandra Barac

Academic Editor, PLoS ONE

Thank you very much for the shift review of this protocol.

We have addressed all the reviewers’ comments and hope this protocol is now acceptable.

Journal Requirements:

Data Availability statement,

**Data Availability:** All relevant data are within the article and its Supporting information files.

Reviewer #1: The proposed article introduces a living systematic review protocol focused on determining the prevalence of Chronic Pulmonary Aspergillosis (CPA) within the continuum of Pulmonary Tuberculosis (TB) care. The protocol is designed to address an important knowledge gap in this area and improve our understanding of the coexistence of CPA and TB in various stages of care.
I find that the research questions are precise, the methodology seems robust and well-structured, and a comprehensive search strategy gives me confidence in the review's ability to provide valuable insights for both researchers and clinicians. Bias and data analysis is clearly outlined and seems robust.
I recommend the publication of this protocol, as it promises to be a good resource that will ultimately improve the care of patients dealing with this condition.

Authors’ response: Thank you very much for commending our protocol.

Reviewer #2: It is an excellent proposal considering that there is a complex interplay between CPA and PTB/Post TB patients.there could be a lot of heterogeneity in the results due to varying sizes of studies, ethnicity, geographic location, timing,population diversity which already has been stated by the authors. For post TB patients, do the authors wish to include those studies only which have done a CT scan and Gene X pert for all the patients?What about immunocompromised patients?ALL in all ,it is a wonderful study and I wish them good luck.

Authors’ response: Thank you very much. We will include both immuncompromised and immunocompetent individuals in whom all the relevant investigations consistent with the diagnostic work up for CPA have been document. We surely appreciate the heterogeneity and complexity of CPA.

Kind regards

Dr Felix Bongomin
